# Artemisinin-Based Antimalarial Drug Therapy: Molecular Pharmacology and Evolving Resistance

**DOI:** 10.3390/tropicalmed4020089

**Published:** 2019-06-04

**Authors:** Laura E. Heller, Paul D. Roepe

**Affiliations:** Departments of Chemistry and of Biochemistry and Cellular and Molecular Biology, Georgetown University, 37th and O Streets NW, Washington, DC 20057, USA; leh58@georgetown.edu

**Keywords:** heme adduct, PfPI3KIII, artemisinin target

## Abstract

The molecular pharmacology of artemisinin (ART)-based antimalarial drugs is incompletely understood. Clinically, these drugs are used in combination with longer lasting partner drugs in several different artemisinin combination therapies (ACTs). ACTs are currently the standard of care against *Plasmodium falciparum* malaria across much of the world. A harbinger of emerging artemisinin resistance (ARTR), known as the delayed clearance phenotype (DCP), has been well documented in South East Asia (SEA) and is beginning to affect the efficacy of some ACTs. Though several genetic mutations have been associated with ARTR/DCP, a molecular mechanism remains elusive. This paper summarizes our current understanding of ART molecular pharmacology and hypotheses for ARTR/DCP.

## 1. Artemisinins and ACTs

**Figure 1:**
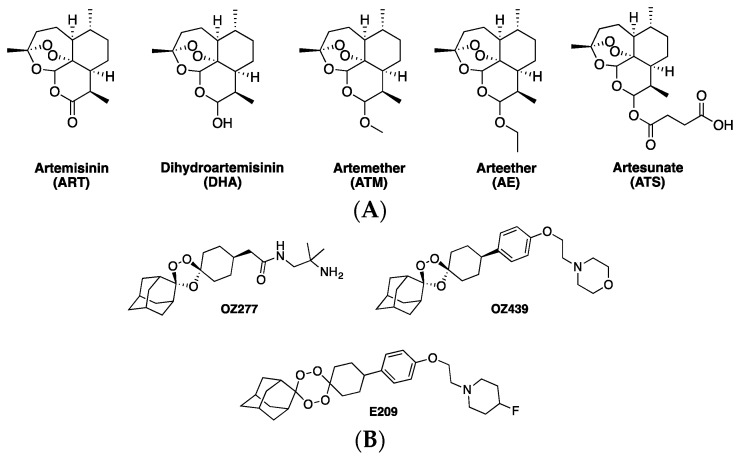
(**A**) Structures of artemisinin (ART) and other clinically viable derivatives. (**B**) Synthetic trioxane and tetraoxane ART analogs (see [2] for OZ277, OZ 439; [3] for E209).

Artemisinin (ART) is a sesquiterpene lactone isolated from *Artemisia annua*, an herb long-employed in traditional Chinese medicine to remedy fevers [1]. Several derivatives of this drug are currently in use for the treatment of malaria disease. These modifications to the ART parent drug improve drug solubility and other characteristics (Figure 1A). Other trioxane and tetraoxane compounds are in various stages of development for use as synthetic alternatives to derivatives of the natural product drug (e.g., Figure 1B). 

ARTs are somewhat unique in their ability to both kill the malarial parasite and inhibit parasite growth at similar very high potency (≤10^−8^ M). These drugs are effective against all intraerythrocytic stages of malarial parasite development, and, in most studies, are particularly potent against the initial ring stage [4]. Depending upon the dose, ring stage parasites treated with ART-based drugs may enter a state of quiescence or dormancy rather than being killed immediately, and they may then resume growth up to weeks after drug treatment [5]. In addition, unlike most other classes of antimalarial drugs, this class also inhibits development of the gametocyte stage of parasite development [6].

ART-based drugs are more precisely viewed as “pro drugs” that must be chemically activated in order to exert their antimalarial effects [7]. The exact mechanism for the activation of the ART endoperoxide pharmacophore (Figure 1) and its relation to parasite death remain an active area of research, as are connections between this chemistry and what appears to be emerging resistance to these drugs [8,9,10]. One popular theory for pro drug activation involves the Fe^2+^-catalyzed Fenton chemistry cleavage of the endoperoxide bridge to generate free radicals, which then, in turn, alkylate a variety of possible intracellular targets, resulting in the death of the parasite [11]. However, a number of details related to pro drug activation and subsequent target alkylation are yet to be elucidated. For example, the source of Fe^2+^ for pro drug activation is not fully defined, although the principle source is likely to be a reduced (ferrous) ferriprotoporphyrin IX (FPIX) heme released during obligate parasite hemoglobin (Hb) catabolism [12]. 

**Figure 2:**
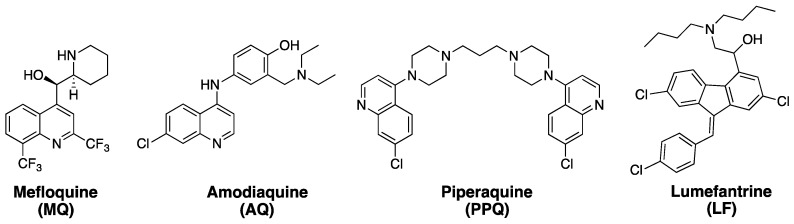
Common ART combination therapies (ACT) partner drugs. The most widely used ACTs are ATM-LF, DHA-PPQ, ATS-MQ, and ATS-AQ.

ART combination therapies (ACTs), which have been widely used since the mid 2000s, are the current front-line therapy for treatment of *P. falciparum* malaria, as recommended by the World Health Organization (WHO) [13]. ART-based drugs have relatively short half-lives (~ 1 h). In ACTs, they are typically paired with a longer lasting “partner” drug with a different mechanism of action (MOA) (Figure 2). Via this strategy, the “parent” ART-based drug often reduces parasite burden in the malarial patient by orders of magnitude within a few hours, and the second “partner” drug then reduces the likelihood of recrudescence and emergence of resistance by killing and/or impeding the growth of the few parasites that remain [14]. In the early 1990s, ATS (Figure 1) was combined with MQ (Figure 2) as an effective ACT, reducing rates of malaria infection significantly along the Thai–Myanmar border [15]. The success of ATS–MQ led to the deployment of other currently popular ACT combinations such as ATM–LF and DHA–PPQ (Figure 1 and Figure 2). 

## 2. Early Investigations of ART Molecular Pharmacology

**Figure 3:**
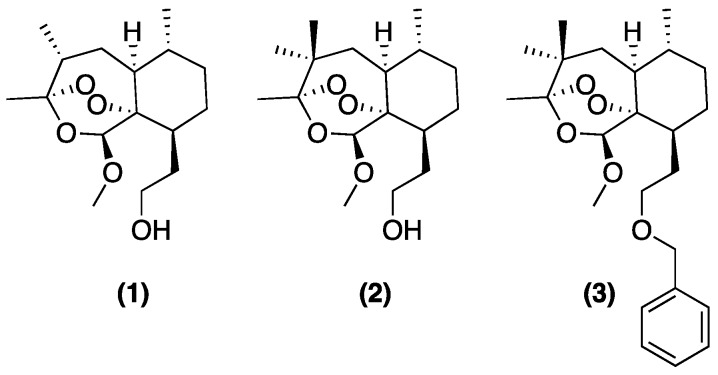
Three methylated ART derivatives with no antimalarial activity, from [24]; see text.

Early on, Meshnick and colleagues treated cultured *P. falciparum* with [^14^C] ART and analyzed the parasite lysate via SDS-PAGE [16]. They concluded that all radioactivity migrated with the gel solvent front, suggesting that ART had bound to target(s) in the parasite, and that the molecular mass of the bound complex(es) was <3000 Da. This result was similar to what was observed when incubating ART with purified FPIX in vitro. Using UV-visible spectroscopy, it was noted that the peak ferric FPIX absorbance at ~400 nm decreased substantially in the presence of DHA. It was also noted that ART pre-incubated with FPIX lowered ART antimalarial potency, while FPIX alone had no activity [16]. The following year, Posner and colleagues monitored the endoperoxide cleavage-mediated breakdown of a tosylated ART derivative (called “5c”) which they had synthesized and labeled with ^18^O [17]. Oxidation clearly led to the cleavage of the endoperoxide bridge. Furthermore, the addition of free iron (II) yielded an oxygen radical, which could be directly monitored via the foresighted placement of ^18^O. Between one and five shifts rearranged the radical to a carbon 4 or “C4” centered radical, which is then capable of alkylating numerous targets. This chemistry was largely characterized by ^13^C and ^1^H NMR experiments and represents the earliest detailed work characterizing the C4 radical for an activated ART-based drug [17]. That same year, Meshnick et al. used cyclic voltammetry to suggest that ART and DHA are irreversibly reduced by incubation with FPIX chloride [18]. Meshnick and colleagues acquired electron paramagnetic resonance (EPR) spectra for ART after 30 min incubation with Fe(II) (in the form of dissolved [NH_4_]_2_Fe[SO_4_]_2_), that suggested the formation of radical species, with no such signal seen for ART or Fe alone [11]. Additional SDS–PAGE data suggested that activated [^3^H]–DHA bound covalently to multiple serum components. In the presence of FPIX, the percent of bound drug increased by 35% and decreased by 10% upon addition of the Fe chelator DFO [19], further supporting a clear link between the ferrous Fe-mediated reduction of ART and covalent drug binding. In 1994, the Meshnick group suggested that activated [^14^C]–ART covalently bound multiple hemoproteins in vitro (e.g., cytochrome c, catalase, methemoglobin, and hemoglobin [Hb]) [20]. By measuring the percent radioactivity bound to purified protein, 5–18% of the added drug was found to be bound to hemoprotein [20]. Since iron that is capable of activating ART-based drugs is found within the FPIX heme, and since copious FPIX is released from Hb within malarial parasites during their intraerythrocytic development [21], Meshnick and colleagues further examined the possibility of an ART–FPIX heme covalent adduct forming in vitro under certain conditions. Mixing FPIX chloride with ART in MeOH and incubating in the dark for 24 h, they found what appeared to be ART–FPIX covalent adduct peaks by mass spectrometry [22]. Additionally, the incubation of [^14^C]–ART with the isolated parasite hemozoin (Hz; crystalline FPIX formed within the malarial parasite digestive vacuole; see [23]) led to a loss of FPIX-associated radioactivity within the supernatant, suggesting that Hz may also bind ART [22]. However, more recent work incorporating additional separation of Hz from FPIX aggregates shows that ART-based drugs do not bind appreciably to Hz, nor do ART drug-FPIX adducts incorporate into growing Hz crystals [12]. Fractionation after the incubation of *P. falciparum*-infected red blood cells (iRBCs) with [^3^H]–ATM revealed small amounts of radioactivity in the supernatant, with ~75% within a 1% SDS wash of the lysed cell pellet [22]. Based on these data, Meshnick and colleagues suggested that [^3^H]–ATM that was perhaps bound to a free FPIX heme was released from the pellet upon treatment with SDS, but no direct measurements of possible drug–heme covalent adducts were performed. To further test the hypothesis that a C4 radical is formed upon the Fe activation of ART, the Meshnick and Posner groups together synthesized ART derivatives with one or two methyl groups added to C4 (Figure 3); such methylated compounds would be unable to form a C4 centered radical. Not surprisingly, the methyl-substituted derivatives showed no antiparasitic activity, further supporting the notion that C4 radical formation was obligatory for the potency of ART pro drugs [24].

**Figure 4:**
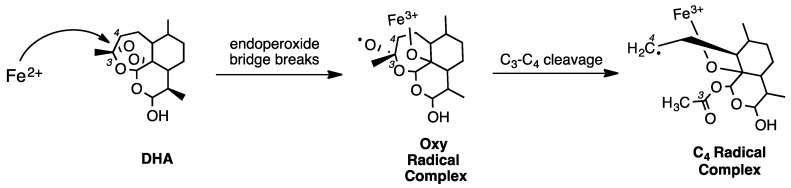
Reaction between ferrous iron and DHA generates an iron–oxo species initially containing oxygen radical, followed by rearrangement to the C4 radical.

Bernard Meunier and colleagues [25] proposed an attractive, chemically logical scheme for radical formation by first studying adducts formed between ART and manganese(II) meso-tetraphenylporphyrin, which could be more easily demetallated and analyzed by NMR spectroscopy, relative to FPIX adduct. Porphyrin–ART adduct data suggested that an O-centered radical is first formed, which then rearranges to a C4 radical upon C3–C4 cleavage (Figure 4). 

**Figure 5:**
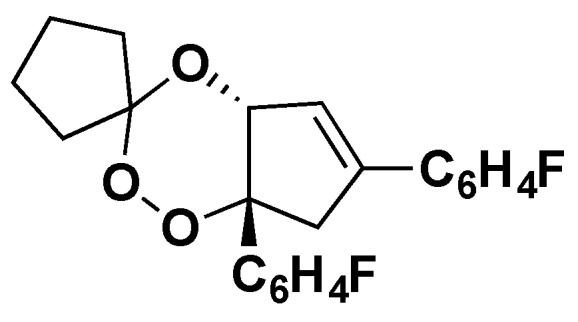
Structure of the endoperoxide “BO7” [26].

Because the radical becomes a flexible terminal alkyl radical, it can easily alkylate many targets via insertion into C–H, C–C, and other bonds. Adducts were also found using ATM and a model endoperoxide compound (BO7; Figure 5) with some similarity to ART [26]. 

**Figure 6:**
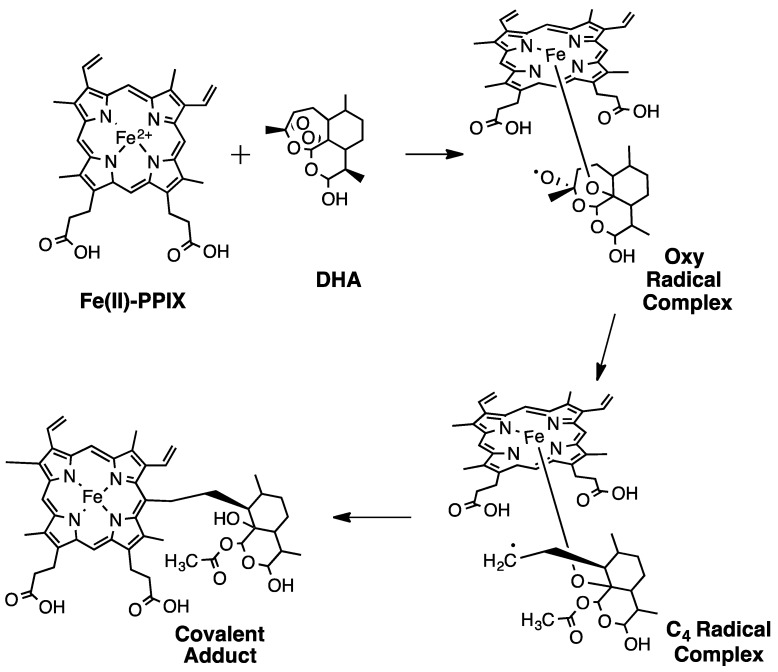
Reaction between ferrous heme (Fe(II)PPIX) and DHA to form a covalent adduct, as initially proposed by [29]; see also [12].

In 2001, Meunier and colleagues monitored the reaction between a reduced heme dimethyl ester and ART dissolved in CH_2_Cl_2_ [27]. While this reaction was much less physiologically relevant, the conditions were useful, since ART is much more soluble in CH_2_Cl_2_. Thus, using NMR spectroscopy, Meunier et al. more easily observed the formation of the oxy radical, the rearrangement to the C4 radical, and the ART alkylation of the FPIX porphyrin in vitro (Figure 6). Based on these results, the following year, Meunier et al. incubated FPIX with equimolar ART and excess glutathione (GSH) for 1 h in (CH_3_)_2_SO and reported the formation of ART–FPIX adducts [28]. Similar adducts were then synthesized and demetallated to remove paramagnetic iron so as to enable closer inspection by NMR. With these experiments, Meunier et al. were able to outline a chemical scheme for the formation of ART radicals (Figure 4) [29]. These experiments were also done with a series of additional endoperoxide compounds, suggesting that adducts can form between these porphyrins and any trioxane drug and that the endoperoxide bridge is an essential element of ART-based drug antimalarial pharmacology [30]. Another group criticized Meunier’s use of (CH_3_)_2_SO as a particularly non-physiologically relevant solvent and suggested that ART derivatives with sterically bulky tails would have lower antimalarial potency due to the tail blocking access to the endoperoxide bridge [31]. However, Meunier and colleagues went on to synthesize additional ART derivatives with bulkier tails and reported antimalarial activity similar to ART, ATS, ATM, and others [32].

Finally, Meunier and colleagues [33] also presented direct evidence for ART-FPIX adduct formation within mice infected with the rodent malarial parasite *P. vinckei* and subsequently treated with ART. Several predicted adduct *m/z* peaks were identified using mass spectrometry (MS), and structures were deduced [33]. These correlated well with MS data for adducts previously synthesized in vitro [22]. Though the location for adduct formation within these mice was not determined in this study, taken along with what is known regarding FPIX heme metabolism during intraerythrocytic development of malarial parasites [21,34,35,36], these early data clearly suggested that the FPIX heme released during parasite catabolism of red blood cell hemoglobin might play an important role in ART-based antimalarial drug pharmacology.

## 3. Proteomics Studies Suggest Multiple ART Drug Targets

Two different groups have recently published the results of proteomics experiments that use derivatives of ART or ART-like trioxane compounds that can be conjugated to biotin using click chemistry to identify protein targets of ART-based drugs. Using high resolution mass spectrometry, these experiments identify many protein targets that are covalently bound to ART after drug endoperoxide cleavage [37,38,39]. For ART probes, Wang et al. [37] synthesized an alkynyl derivative of ART which they then “clicked” to a biotin azide via copper catalyzed cycloaddition, while Ismail et al. [38] synthesized a similar molecule as well as an azide derivative of ART which could be clicked to an alkynyl biotin. Wang et al. identified 124 potential protein targets to which their ART analog bound; Ismail et al. found 49 protein targets, as well as 62 targets for a non-ART trioxane probe [37,38,39]. Importantly, unlike the FPIX heme target identified for ART drugs described above [12], the levels of labeled versus unlabeled ART targets are not quantified in these studies, nor are any differences in labeling for ARTS versus artemisinin resistance (ARTR) parasites inspected. Regardless, when comparing the results of these three proteomics studies, it is interesting to note that only 19 proteins are commonly alkylated in all three studies, suggesting that most ART-protein adduct formation is somewhat random. Listed below along with their putative locations within the *P. falciparum* parasite are the proteins that are labeled in all three studies (Table 1). Notable protein targets that covalently bound to all three ART probes include plasmepsins I and II and multidrug resistance protein (MRP), all of which are localized to the parasite DV. Some bias towards labeling DV localized proteins might be expected since the DV is the site of FPIX release and, hence, the site of FPIX-mediated ART drug activation [40]. 

Altered gene expression was also examined, and genes that are mildly up- or down-regulated at the ring or trophozoite stages are shown in Table 2. These include genes that encode DV localized proteins such as plasmepsin I. Of the eight genes found alternately expressed in all three studies, five (merozoite surface protein I, tubulin beta chain, actin I, l-lactate dehydrogenase, and plasmepsin I) are essential. A particularly pharmacologically relevant protein target would be expected to be found in all three drug target proteomics studies and to be an essential protein, and the gene that encodes the protein might be expected to be down-regulated after drug exposure. The only protein that meets all three of these criteria and is also localized to the DV is plasmepsin I. Interestingly, plasmepsin I is expected to be proximal to the ART-activating FPIX heme that is being released from catabolized Hb, because it is during the plasmepsin I and II catalyzed steps of Hb proteolysis that the FPIX heme is released from Hb. ART-based drugs are clearly capable of alkylating multiple intra parasite targets once activated by a ferrous heme; we suggest that targets that are both in the highest abundance and closest to the site of FPIX release from catabolized Hb are the most likely to be alkylated and, hence, the most relevant for further elucidating ART-based drug molecular pharmacology and ARTR. Interestingly, additional plasmepsin isoforms (plasmepsins II and III) have also been associated with resistance to the ACT companion drug piperaquine (PPQ) [41]. 

## 4. Initial Evidence for Evolving ART Resistance (ARTR)

ART combination therapies (ACTs) were introduced in response to reduced efficacy of multiple antimalarial drugs including CQ, SP, and MQ, and, by 2006, were the WHO-recommended treatment for *P. falciparum* malaria worldwide [42]. Unfortunately, a harbinger of evolving ART resistance (ARTR), also known as the delayed clearance phenotype (DCP) [8,9,10] has been documented in western Cambodia and has spread to the Greater Mekong Subregion, a transnational area of the Mekong River basin in Southeast Asia. In 2002, the 28-day failure for the ACT ATS–MQ in the Pailin district of western Cambodia was ~14%, but, just two years later, 42-day failure rates of up to 21% were documented [43]. ATS–MQ failure was also seen in the Trat province within Thailand, close to its border with Cambodia [44]. Initially, resistance to MQ alone was thought to be the origin of these clinical failures, since MQR had been documented in this region for some time [45,46]. However, at this time, somewhat lower cure rates were also being observed upon ATM–LF ACT use in the nearby Battambang Province of Cambodia [47]. To examine these troubling trends in more detail, a controlled study was performed wherein ATS was clinically administered as a monotherapy, and parasite clearance was closely monitored [48]. Despite acceptable plasma drug levels, parasite clearance rates were twice as slow for malarial patients from Cambodia relative to the Thai–Myanmar border. This delay in parasite clearance (DCP) has subsequently been found to be growing and increasingly common across mainland and Southeast Asia [48]. This DCP phenotype has been observed after treatment with the ACT DHA–PPQ as well, where DHA–PPQ failure may be caused by evolving resistance to both DHA and PPQ [49].

Due to these examples of slow parasite clearance noted after ART-based monotherapy or ACT treatment, a series of genome-wide association studies (GWAS) were done to search for a genetic basis for what was assumed to be evolving ARTR (DCP). It soon became clear that ARTR is heritable [50], and that it is associated with multiple founder populations [51]. GWAS initially detected a region on *P. falciparum* chromosome 13 that was strongly associated with DCP [52]. Subsequently, parasites cultured in the laboratory under intermittent ART pressure for five years were sequenced [53], and a mutation within a gene on chromosome 13 was found [54,55]. This gene encodes a protein of unknown function that harbors a Kelch motif now known as “PfKelch13” or “PfK13”. A large number of Cambodian isolates were found to contain non-synonymous point mutations in the region of the *PfK13* gene that encodes the PfK13 Kelch propeller domain. It was hypothesized that *P. falciparum* parasites harboring PfK13 Kelch domain amino acid substitutions were better able to survive initial ACT drug exposure, which over time might then also lead to ACT partner drug resistances and, eventually, an overall decline in ACT efficacy [56]. The race was on to better define evolving ARTR, since, other than current ACTs in use, no other currently approved drug combinations are efficacious across the globe for treating all drug resistant malaria. Additional monitoring as well as a more complete molecular definition of ARTR/DCP are now both desperately needed. 

## 5. Hemoglobin (Hb) and Glutathione (GSH) Metabolism Versus ART Potency

Since it is clear that ART-based drugs are highly reactive in the presence of a reduced FPIX heme, since parasites digest nearly all host red blood cell Hb releasing large amounts of free FPIX within the parasite digestive vacuole (DV), and since GSH likely maintains ferrous FPIX pools within the DV [57,58,59], attention has focused on any possible connection between the loss of ART potency and altered Hb or GSH metabolism in ARTR parasites, as well as perhaps other changes that could, in theory, affect levels of the reduced FPIX heme within the parasite. The Tilley group [60] studied the relationship between Hb metabolism and ART potency, and, after measuring fluorescein–dextran uptake (as a surrogate of Hb uptake) by parasites upon treatment with increasing [ART], suggested that drug treatment inhibits parasite endocytosis of host RBC cytoplasm. Testing the theory that Hb digestion is required for the antimalarial activity of ART-based drugs, Tilley and colleagues treated CQ and ART sensitive 3D7 parasites with ART drugs in the presence of the cysteine protease inhibitor *N*-acetyl-l-leucyl-l-leucyl-l-norleucinal (ALLN). The addition of ALLN appeared to minimize the effect of ART drugs, with up to 90% parasite survival even in the presence of 1 mM ART [60]. Relatedly, for 3D7 parasites harboring deletions in the Hb protease falcipain 2, ART was found to be less potent: A four-hour bolus dose of 800 nM ART left 20% of parasites viable, compared to 2–5% for unmodified 3D7. These data again support the theory that abundance of the free FPIX heme (a direct consequence of parasite Hb metabolism) is essential for ART pro drug activation and the potency of ART-based drugs. 

Recently, Heller et al. quantified the abundance of free FPIX and Hz at various stages of the *P. falciparum* life cycle for ARTS versus ARTR parasites [40]. As previously noted by Kyle and colleagues [61], ARTR parasites showed slightly longer ring stage development times and shortened trophozoite stages relative to ARTS parasites. Not surprisingly, Heller et al. also found altered levels of free FPIX at specific time points during the ARTR parasite life cycle relative to matched isogenic ARTS control [40]. Interestingly, the level of total free FPIX was found to be lower for ARTR parasites at the trophozoite stage but slightly higher at the ring and schizont stages of development. ARTR parasites also showed slightly faster and elevated levels of Hz production from free FPIX [40]. As described, because ART-based drugs are activated by free FPIX, Heller et al. also searched for potential ART–FPIX adducts formed within parasites after treatment with plasma levels of DHA. DHA–FPIX adducts were extracted and quantified by mass spectrometry [12], and, not surprisingly, the largest abundance of adduct was found at the trophozoite stage, corresponding to the stage that harbors the highest concentration of the free FPIX heme within the parasite DV. Importantly, ARTR parasites showed a lower adduct abundance relative to ARTS parasites at all stages of parasite development. Since it is a reduced heme, not a total heme, that is relevant for activating ART pro drugs, these data suggest lower levels of reduced FPIX for all ARTR stages, including at the ring and schizont stages [12]. 

The Tilley group tested additional effects of iron chelators on ART potency [62]. Treating ARTS 3D7 parasites with BiPY (an Fe^2+^ specific chelator, 2,2′-bipyridyl) or DFP (Deferiprone, specific for Fe^+3^ chelation) showed that these iron chelators have mild but antagonistic effects on ART and DHA. The influence of the cysteine protease inhibitor, E64d, on ART potency was also examined, and, similar to earlier studies with falcipain mutants, pre-incubation with E64d to inhibit cys proteases was necessary for parasite Hb catabolism-inhibited ART potency. Interestingly, antagonism was most pronounced in the early ring stages, further identifying *P. falciparum* falcipains as active during the ring stage of development and underscoring the ring stage effects of ART-based drugs.

In addition to Hb metabolism, ART drug activation and potency likely depends upon levels of GSH within the parasite, particularly within the DV [12]. It is reduced FPIX (ferrous or Fe^2+^ heme) that will activate ART drugs, not the ferric heme, and, although other mechanisms are possible, GSH is the most biologically-likely reductant for maintaining Fe^2+^FPIX pools within the DV. Fe^2+^FPIX initially released during parasite Hb catabolism within the DV is oxidized to the ferric (Fe^3+^) heme, thus GSH/GSSG ratios within the DV will control the ferrous/ferric heme ratios and hence the relative degree of ART pro drug activation. GSH is synthesized de novo by gamma glutamylcysteine synthetase (ggcs). Previously, *P. berghei* parasites resistant to the quinolinal drugs CQ or MQ showed an increase in *P. berghei* ggcs [63]. To study the relationship between GSH levels and ART drug potency, these investigators disrupted or overexpressed *P. berghei* ggcs [64]. *Pbggcs* mRNA levels for *ggcs* overexpressors were ~5 times higher than that of control wild type parasites, however. Though differences in recrudescence within the mouse malaria model were observed (see below), the presumed increased level of glutamylcysteine synthetase accompanying increased mRNA did not appear to effect parasite growth in culture or laboratory ED_50_ versus CQ or ART via conventional assays. It is possible that altering the level of only one of several enzymes that affect GSH/GSSG cycling is insufficient for altering [GSH] within the DV to an extent that would influence drug response in culture. Additional direct measurements of this key metabolite within *P. falciparum* are needed. 

**Figure 7:**
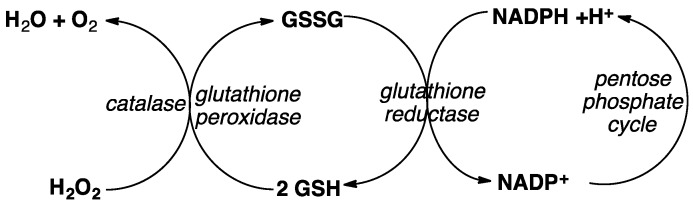
Enzyme catalyzed reactions contributing to glutathione (GSH)/GSSG cycling in living cells.

On the other hand, after dosing mice infected with wild type *P. berghei*, parasites overexpressing *Pbggcs*, or a *Pbggcs* null strain with 20 m/kg DHA per day for four days, recrudescence was observed within 2–3 days for wild type and *Pbggcs* overexpressors, but mice infected with *Pbggcs* null parasites did not show recrudescence. These results suggest that perturbation in GSH levels influences parasite response to ART treatment in vivo. Since GSH/GSSG cycling is controlled by multiple enzymes (Figure 7), the manipulation of only one gene contributing to this cycling is unlikely to reveal a direct correlation with GSH levels in multiple cellular compartments. Much more detailed work on this point remains to be done. 

## 6. Additional Evidence for Altered Drug-FPIX Interactions in ARTR Parasites

A recent metabolomics study [65] identified differences for ARTS versus ARTR field isolates, including the up-regulation of part of the “unfolded protein response” (UPR) pathway in ARTR parasites. Mok et al. also found up-regulation of Hb digestion in ARTR schizont stage parasites and the down-regulation of GSH metabolism and of Hb digestion in ARTR trophozoite stage parasites relative to ARTS. While Mok et al. used field isolates in these studies, which are typically difficult to culture, a more recent paper by the same group [66] derived two laboratory ARTR strains by pressuring early ring stage 3D7 (ARTS) parasites with 900 nM ART for four hours every other parasite life cycle, for approximately 1.3 yrs. Though this drug pressure does not mimic what typically occurs within malarial patients, the results were nonetheless informative. Whole genome sequencing was done to identify mutations that distinguished laboratory-derived ARTR from ARTS strains. The DV localized hemoglobinase falcipain 1 was observed to be up-regulated across the parasite life cycle in the ARTR parasites relative to ARTS. Table 3 and Table 4 highlight genes specifically up- or down-regulated in the mid to late ring stage of laboratory derived ARTR relative to ARTS parasites [66]. Interestingly, and as predicted by models that envision the FPIX heme activation of ART-based drugs, multiple enzymes whose collective activities influence GSH/GSSG ratios, and, hence, ferrous/ferric FPIX ratios, are found to be altered. It should prove interesting to further quantify the relative effects of up regulated GSH synthase (Table 3) versus down-regulated glutathione reductase (Table 4), which are predicted to increase and decrease the availability of GSH, respectively, along with companion direct measurements of [GSH]. 

## 7. *PfK13* Mutations Associated with ARTR

As described above, molecular markers for at least one form of ARTR/DCP have been identified. These are non-synonymous single nucleotide polymorphisms (SNPs) in the *PfPK13* gene that confer amino acid substitutions in the Kelch domain propeller region of the encoded PfK13 protein [48,53]. Throughout the field of drug resistance research, the degree of drug resistance, as well as any pattern in resistances to multiple drugs, is typically quantified by ratio’ing the measured potency of the relevant drug(s) in resistant versus sensitive cells. For antimalarial drug resistance, even though an arguably more clinically relevant measure of antimalarial drug potency is drug LD_50_ (cytocidal potency), drug IC_50_ values, or cytostatic potency (the concentration of drug required to inhibit parasite growth by 50%), are typically used to calculate “fold” drug resistance [67]. Importantly, however, with a few exceptions, the ARTR malarial parasites discussed above do not show easily measureable shifts in either ART drug LD_50_ or IC_50_. One exception is a study that reports small shifts in LD_50_ for the ring stages of a laboratory strain of ARTR parasites [62]. Thus, Witkowski and colleagues developed a specialized assay known as the RSA (ring stage assay) to quantify ARTR [68]. In the RSA, early ring-stage parasites are bolus-dosed with clinically relevant plasma levels of drug (typically, 10^−7^–10^−6^ M DHA) for 6 h. The parasites are then washed and cultured in drug-free media, and the relative outgrowth is determined 66 h later. Differences can be quantified to calibrate the degree of resistance to multiple ART-based drugs [69]. ARTS parasite strains yield an RSA outgrowth of ~0–1% compared to ≥10% for ARTR under typical RSA conditions. The basis of the difference in outgrowth is not entirely clear, but a popular hypothesis for the RSA behavior of ARTR versus ARTS is that ART drug potency is highest for ring stage parasites, and that ARTR parasite ring stages show different ART drug-induced quiescence. The RSA has been used to show that introducing PfK13 amino acid substitutions into ARTS parasites by reverse genetics, without ART drug selection pressure, causes ARTR [70]. Similarly, removing the *PfK13* mutation from clinical isolates and introducing an alternate PfK13 amino acid substitution also causes ARTR, as determined by RSA [70]. Interestingly, introducing the same PfK13 propeller mutations into different parasite genetic backgrounds appears to result in variable RSA outcomes, suggesting that, while PfK13 mutations are necessary to explain at least common forms of ARTR, these mutations are not sufficient to account for all observed phenotypes [70]. Since, to our knowledge, all ARTR field isolates contain only one amino acid substitution in PfK13, it is also possible that the position and properties of the substituted amino acid affect in vitro and in vivo phenotypes. Additionally, similar to other antimalarial drug resistance phenomena, it is possible that compensatory fitness mutations, in response to PfK13 amino acid substitutions, further influence evolving ARTR phenotypes. In summation, while it is now widely accepted that non-synonymous *PfK13* mutations are an important ARTR marker, it is likely that other molecular mechanisms either add to PfK13 effects in conferring ARTR or may confer additional (non-PfK13-mediated) ARTR phenomena [71,72].

## 8. PfPI3K

A study by the Haldar group [73] suggested that ART drugs inhibit the sole *P. falciparum* phosphatidyl-3′-kinase (PfPI3K/PfVps34) through non covalent drug–enzyme interactions, and that altered drug–PfPI3K interaction was the basis of ARTR. This study reported that ARTR parasites showed modestly increased levels of PI3P (~1.5 to 2-fold) for both clinical isolates and laboratory engineered strains expressing C580Y PfK13 linked to ARTR. A correlation between levels of PI3P and ARTR as measured by the ring stage survival assay (RSA) was reported, suggesting that PI3K activity may be linked to a decreased sensitivity to ART compounds in mutant PfK13 parasite strains (PfPI3K is a class 3 PI3K enzyme that converts PI to PI3P [74]). Based on results of co-immunoprecipitation experiments, Mbengue et al. hypothesized that the modest increase in PI3P for ARTR parasites is due to diminished C580Y mutant PfK13 binding to PfPI3K and, hence, a diminished ubiquitinylation-dependent degradation of the kinase, thereby acting to increase levels of the PI3P lipid. Additional effects of PfPfK13 on PI3P levels were recently measured [75]. Parasites expressing ARTR-linked C580Y were found to have elevated levels of ER-derived PI3P positive vesicles that migrated through the parasite and were exported to the RBC. The authors hypothesize that PI3P-containing vesicles are amplified in very early rings (0–3 HPI [hours post invasion]) for PfPfK13 mutant parasites [75].

In these studies, the DHA binding site presumed to lie within PfPI3K was modeled using molecular dynamics simulations, and Mbengue et al. proposed binding that relies on hydrogen bonding between the enzyme and an intact endoperoxide group on the ART drug. However, as described above, a cleavage of the DHA endoperoxide bridge is necessary for antimalarial potency and hence interaction with pharmacologically relevant targets of ART-based drugs. Thus, Hassett et al. further studied the binding of ART drugs to purified PfPI3K [74]. PfPI3K lacks pleckstrin homology domains and Ras binding sites that are characteristic of class I and II PI3Ks [76] and most closely resembles Vps34, the sole class III PI3K, [77,78]. Hassett et al. purified PfVps34 that was heterologously expressed in yeast after *PfVps34* codon optimization; they then measured PI3P production from PI for the purified enzyme [79]. Inhibition by ART drugs and related ozonide compounds was also quantified [74], and no effect on PfVps34 activity was found in the absence of ferrous iron (either in the form of a ferrous FPIX heme or FeSO_4_). However, in the presence of iron, DHA inhibited Vps34 activity at nM concentrations and bound covalently to the enzyme. The direct mapping of covalent drug binding sites by mass spectrometry showed that the endoperoxide cleavage of ART drugs is indeed necessary for the inhibition of PfPI3K, and that binding is somewhat random (i.e., not to one specific binding site). In addition, the use of ART drug EC_50_ for PfVps34 inhibition did not appear to correlate with antiplasmodial potency (R^2^ = 0.54), further suggesting that PfVps34 is not the primary, or not the only, pharmacologically relevant ART-based drug target within *P. falciparum* parasites. 

As is the case for all other eukaryotic Vps34 enzymes, PfVps34 plays a role in regulating autophagy, which is both an important cellular survival pathway and a pathway that can influence the extent of drug-induced cell death [80,81]. Interestingly, a recent GWAS identified a locus on chr10 encoding another autophagy regulator (PfAtg18) as being associated with sensitivity to DHA and ATM in some ARTR isolates [82]. PI3K inhibitors have been found to be potent antimalarial compounds that inhibit the parasite autophagy-like pathway and that are also synergistic in combination with ART-based drugs [69,83]. Lastly, no obvious mTOR orthologue is found for *P. falciparum* [84], yet mTOR kinase activity is typically a key step in initiating autophagy, further suggesting that PfVps34 may be a particularly critical regulator of the *P. falciparum* drug or starvation-induced autophagy pathway. We suggest that PfVps34 plays an important role in the parasite’s response to ART-based drugs through its effect on regulating drug-induced parasite cell death mediated by the parasite autophagy-like cascade [80]. 

## 9. ARTR without Associated *PfK13* Mutations

As alluded to above, several recent studies suggest that ARTR/DCP phenotypes can evolve without concomitant PfPfK13 amino acid substitutions. Between 2015 and 2016, Sutherland et al. genotyped parasites isolated from four cases of recurrent *P. falciparum* malaria in patients treated with ACT in Angola, Uganda, and Liberia. The recrudescent parasite isolates from each patient harbored PfK13 mutations at several positions; however, no non-synonymous mutations were found in the propeller-encoding domain of the *pfk13* locus. This suggests that *pfk13*-independent mechanism(s) are involved in altered recrudescence for these African isolates. One interpretation is that ARTR without PfK13 Kelch domain amino acid substitutions can occur. Another interpretation is that, since these isolates were not analyzed for RSA phenotypes, it is possible that a clinical observation of DCP can occur without PfK13 mediated ARTR. 

In 2018, Demas et al. carried out a long-term in vitro selection, yielding ARTR parasites that also did not show propeller domain PfK13 mutations. By exposing ARTS Senegalese parasite isolate-derived strains to 13 selection cycles of increasing concentrations of DHA for 48 h periods, two ARTR laboratory-selected strains were isolated. These both showed mean RSA survival percentages similar to those found for PfK13 C580Y-harboring strains. Sequencing of the PfKelch13 locus for the selected Senegalese strains revealed no genetic changes. Instead, whole genome sequencing identified 10 different mutations in several genes, and PfCoronin mutations were found to be correlated with laboratory derived ARTR [72]. To our knowledge, these mutations have, to date, not been found in field isolates. 

ART exposure has also been linked to a dormancy phenomenon that seems unrelated to PfK13 mutations [85]. In this work, cultured parasites were exposed to drugs, survived as non-progressing rings, and then eventually recrudesced weeks later. Breglio and colleagues sought to determine if the ability of parasites to withstand ART exposure by existing in a dormant state was associated with PfK13 mutations. Studying two Cambodian field isolates, they determined that recrudescence from dormancy was similar for wild type parasites versus those harboring either ARTR-associated R539T or C580Y PfK13 mutations. Thus, these PfK13 mutations, sufficient to confer ARTR as measured by other assays [70], were not necessary for the dormancy phenotype examined in Breglio et al. [85]. 

In order to further probe ARTR phenotypes, Sa et al. performed a genetic cross between wild type PfK13 C580 and mutant PfK13 Y580 parasite strains, isolated progeny, and performed QTL analysis versus several phenotypic features [86]. An increased ring stage survival after bolus dosing parasites with DHA [68] was linked to C580Y inheritance; however, recrudescence was not linked to the C580Y mutation in this study, similar to results found by Sutherland et al. [71] and Breglio et al. [85]. There was also no significant difference between in vivo parasite clearance half-times for *Aotus* monkeys infected with C580 versus C580Y strains. Thus, while RSA differences are observed between wild type and PfK13 mutant parasites, these additional data suggest that monitoring for PfK13 mutations alone may not be sufficient for full understanding of evolving ARTR/DCP phenotypes. 

## 10. Conclusions

An evolving resistance to ART-based antimalarial drugs threatens many millions around the globe. The elucidation of ART drug molecular pharmacology as well as the molecular mechanism of ARTR are now top priorities in the ongoing world-wide battle against *P. falciparum* malaria. Rapid and considerable recent progress, along with earlier work defining the chemistry of ART-based drugs, now defines a list of research priorities. 

## Figures and Tables

**Table 1:** Protein targets of ART-based drugs found in all three proteomics studies discussed in the text [37,38,39].

Protein (Gene) ID	Prtotein ART Targets Found in All 3 Studies	Putative Location
PF3D7_0102200	Ring-infected erythrocyte surface antigen	Dense Granules (Merozoites)
PF3D7_0322900	40S ribosomal protein S3A, putative	Ribosome
PF3D7_0523000	Multidrug resistance protein	**DV**
PF3D7_0608800	Ornithine aminotransferase	Cytosol
PF3D7_0624000	Hexokinase	Cytosol
PF3D7_0708400	Heat shock protein 90	Cytoplasm
PF3D7_0818900	Heat shock protein 70	Nucleus
PF3D7_0903700	Alpha tubulin 1	Microtubule
PF3D7_0930300	Merozoite surface protein 1	Plasma Membrane
PF3D7_1008700	Tubulin beta chain	Microtubule
PF3D7_1012400	Hypoxanthine-guanine phosphoribosyltransferase	Cytosol
PF3D7_1015900	Enolase	**DV**
PF3D7_1246200	Actin I	Actin Filament/Cytoskeleton
PF3D7_1311900	Vacuolar ATP synthase subunit a	**DV**
PF3D7_1324900	L-lactate dehydrogenase	Cytosol
PF3D7_1357100	Elongation factor 1-alpha	Cytosol
PF3D7_1407900	Plasmepsin I	**DV**
PF3D7_1408000	Plasmepsin II	**DV**
PF3D7_1444800	Fructose-bisphosphate aldolase	Cytosol

**Table 2:** Top hits for up- or down-regulated genes found in all three studies described in the text. Log 2 expression values are shown, where a value of 0 indicates normal expression, and positive or negative values denote up- or down-regulation, respectively. Genes with values more than +/− 0.75 are shown in italics, and those with values more than +/− 1.25 are shown in bold.

Gene ID	Found in All 3 Studies	Putative Location of Encoded Protein	Ring (9 HPI) Expression Value (Log2 Ratio) in 3D7	Trophozoite (28 HPI) Expression Value (Log2 Ratio) in 3D7	Essential?
PF3D7_0102200	Ring-infected erythrocyte surface antigen	Dense Granules (Merozoites)	*0.8*	**−2.52**	Unknown
PF3D7_0608800	Ornithine aminotransferase	Cytoplasm	**−1.74**	0.7	No
PF3D7_0903700	Alpha tubulin 1	Microtubule	**−1.3**	−0.08	No
PF3D7_0930300	Merozoite surface protein 1	Plasma Membrane	−0.59	**−1.52**	**Yes**
PF3D7_1008700	Tubulin beta chain	Microtubule	**−1.53**	−0.02	**Yes**
PF3D7_1246200	Actin I	Actin Filament/Cytoskeleton	−0.55	*−1.05*	**Yes**
PF3D7_1324900	L-lactate dehydrogenase	Cytosol	*−0.76*	0.49	**Yes**
PF3D7_1407900	Plasmepsin I	**DV**	*1.17*	*−1.01*	**Yes**

**Table 3:** Genes up-regulated in artemisinin resistance (ARTR) parasites as observed by Rocamora et al. [66].

Gene ID	Up-Regulated Gene Description	FDR
PF3D7_0512200	glutathione synthetase	0.20
PF3D7_1224600	cytochrome c heme lyase, putative	0.23
PF3D7_0825600	cytochrome c oxidase assembly protein, putative	0.19
PF3D7_1311700	cytochrome c2 precursor, putative	0.21

**Table 4:** Genes down-regulated in ARTR parasites as observed by Rocamora et al. [66].

Gene ID	Down-Regulated Gene Description	FDR
PF3D7_0727200	cysteine desulfurase, putative	0.19
PF3D7_1438900	thioredoxin peroxidase 1	0.24
PF3D7_1457200	thioredoxin 1	0.12
PF3D7_1455400	hemolysin, putative	0.22
PF3D7_1419800	glutathione reductase	0.16
PF3D7_1012300	ubiquitinol-cytochrome c reductase complex	0.19
PF3D7_1352500	thioredoxin-related protein, putative	0.24
PF3D7_1011900	heme oxygenase	0.24
PF3D7_1458000	cysteine proteinase falcipain 1	0.16

## Abbreviations

ACT	artemisinin combination therapy
ALLN	*N*-acetyl-L-leucyl-L-norleucinal
AQ	amodiaquine
ART	artemisinin
ARTR/S	artemisinin resistant/sensitive
ATM	artemether
ATS	artesunate
BiPY	2,2′-bipyridyl
DCP	delayed clearance phenotype
DFO	Deferoxamine
DFP	Deferiprone
DHA	Dihydroartemisinin
DV	digestive vacuole
EPR	electron paramagnetic resonance
ER	endoplasmic reticulum
FPIX	ferriprotoporphyrin IX heme
ggcs,	gamma glutamylcysteine synthetase
GSH	glutathione
GSSG	glutathione disulfide
GWAS	genome-wide association study
Hb	hemoglobin
Hz	hemozoin
IC_50_	half-maximal inhibitory concentration
iRBC	infected red blood cell
LD_50_	half-maximal lethal concentration
LF	lumefantrine
MOA	mechanism of action
MQ	mefloquine
MRP	multidrug resistance protein
MS	mass spectrometry
PfPI3K	*Plasmodium falciparum* phosphatidyl-3′-kinase
PPQ	piperaquine
RSA	ring stage assay
SDS-PAGE	sodium dodecyl sulfate-polyacrylamide gel electrophoresis
SEA	Southeast Asia
SNP	single nucleotide polymorphism
QTL	quantitative trait locus
